# Single Leaflet Device Attachment After Pascal Implantation for Transcatheter Edge-to-Edge Repair: Systematic Review and Meta-Analysis

**DOI:** 10.1016/j.shj.2025.100698

**Published:** 2025-07-05

**Authors:** Soumya Gupta, Devika Aggarwal, Michael Gao, Kirtipal Bhatia, Marija Petrovic, Abel Casso Dominguez, Stamatios Lerakis, Edgar Argulian

**Affiliations:** a Department of Internal Medicine, Mount Sinai Morningside/West, New York, New York, USA; b Division of Cardiology, Mount Sinai Fuster Heart, Mount Sinai Morningside Hospital, New York, New York, USA; c Department of Medicine, Icahn School of Medicine at Mount Sinai, New York, New York, USA; d Division of Cardiology, Montefiore Medical Center/Albert Einstein College of Medicine, Bronx, New York, USA; e Division of Cardiology, Mount Sinai Fuster Heart, Mount Sinai Hospital, New York, New York, USA

**Keywords:** MitraClip, Mitral regurgitation, Pascal device, SLDA, Transcatheter edge-to-edge repair

Transcatheter edge-to-edge repair (TEER) has become a standard treatment for patients with primary mitral regurgitation (MR) who are at high or prohibitive surgical risk and for selected patients with secondary MR with persistent symptoms despite optimal medical therapy.^1^ While the widely used MitraClip (Abbott, Santa Clara, California) has been the primary device for this procedure, the novel PASCAL device (Edwards Lifesciences, Irvine, California) has been introduced as an alternative. Single leaflet device attachment (SLDA) though infrequent, is the most common device-related complication, contributing to recurrent MR and increased risk of heart failure hospitalizations and mortality. Data exploring the incidence of SLDA and procedural success rates among patients receiving the PASCAL device are limited. This meta-analysis seeks to address this gap by systematically evaluating pooled SLDA incidence rates as well as the procedural success rate across published studies including primary or secondary moderate-to-severe MR.

We conducted a literature search of Medline, EMBASE, and Cochrane CENTRAL databases from inception to June 27th, 2024. Both observational studies and randomized trials were included if they consisted of patients >18 years with primary or secondary MR undergoing TEER with a PASCAL device and reported rates of SLDA or procedural success rates. Procedural success was defined as successful placement of a PASCAL device (either PASCAL ACE or P10) with resulting reduction in MR to ≤2+. The primary outcome of interest was the incidence of SLDA during follow-up and the secondary outcome was procedural success rate.

Using Freeman-Tukey transformation to estimate effect sizes, we calculated the weighted incidence of SLDA and procedural success using the random effects restricted maximum likelihood model.^2^ Confidence interval (CI)s were calculated using the Wilson’s method. Heterogeneity among studies was assessed using the Higgins I^2^ value.^3^ A random-effects meta-regression analysis was conducted to analyze the effect of female sex and secondary or mixed mechanism of MR on the incidence of SLDA. Reported *p* values were two-tailed with statistical significance specified at 0.05. Statistical analyses were conducted using Stata 18 (Stata Corp, College Station, TX).

We identified 13 studies including 1111 patients that underwent TEER with a PASCAL device. The average age was 78 years, and 41% were females. In terms of mechanism, 64% had functional or mixed MR, while 36% had primary MR. The pooled incidence of SLDA was 1.0% (95% CI: 0.4%-1.9%, I^2^ = 0.01%) (Figure 1a). Among studies where at least 50 patients were included, incidence of SLDA was 1.4% (95% CI 0.6%-2.5%, I^2^ = 0%). All SLDA events occurred either peri-procedurally or within 30 days of follow-up, with most patients requiring another PASCAL device placement, while one patient underwent surgical reintervention. The procedural success rate was 95.0% (95% CI: 94.0%-96.0%, I^2^ = 0%) (Figure 1b). Analysis of the funnel plot suggested no significant publication bias (Figure 1c). Meta-regression showed no significant effect modification of sex (*p* = 0.56) or secondary or mixed mechanism of MR on the estimated pooled SLDA rates (*p* = 0.73).

**Figure 1:**
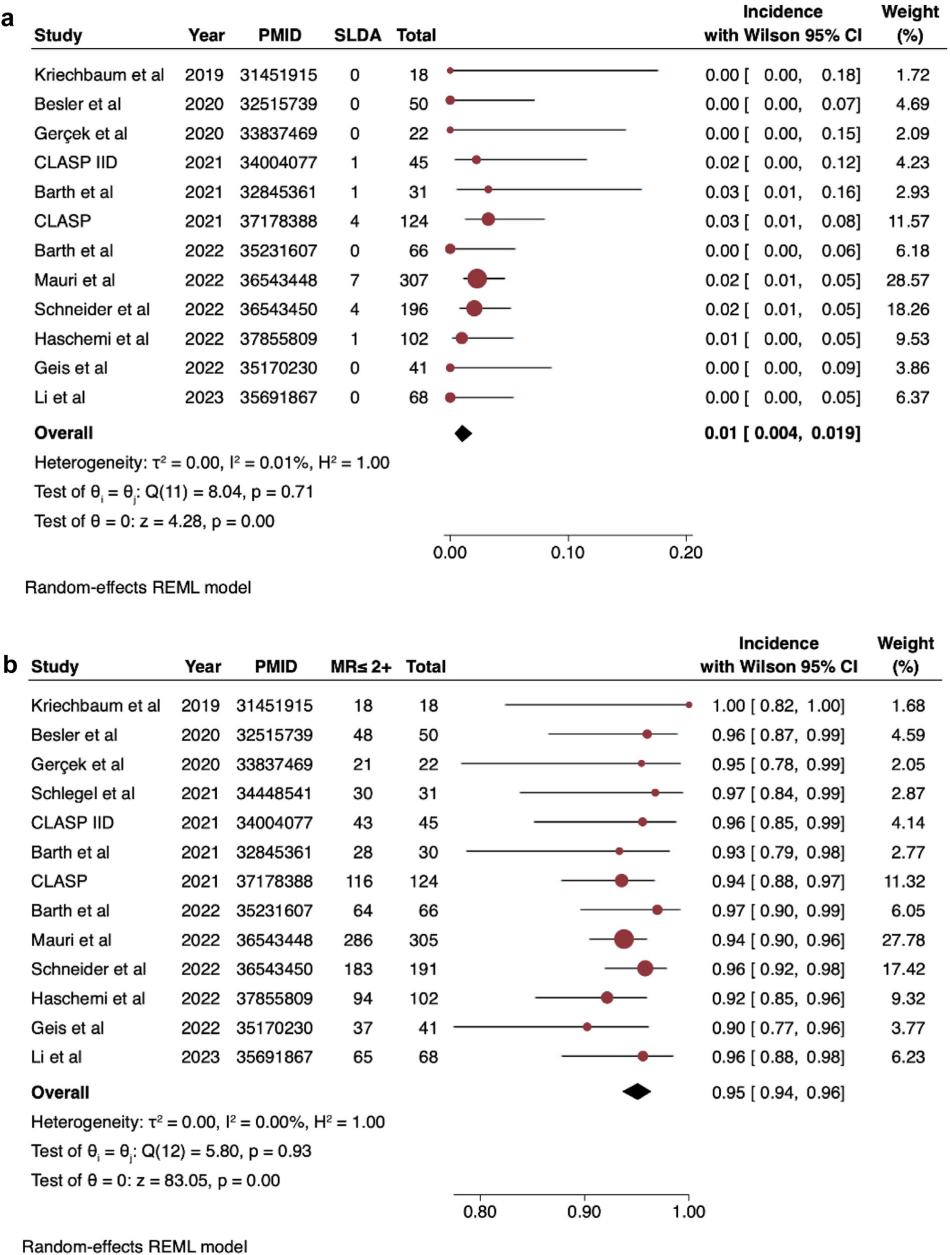

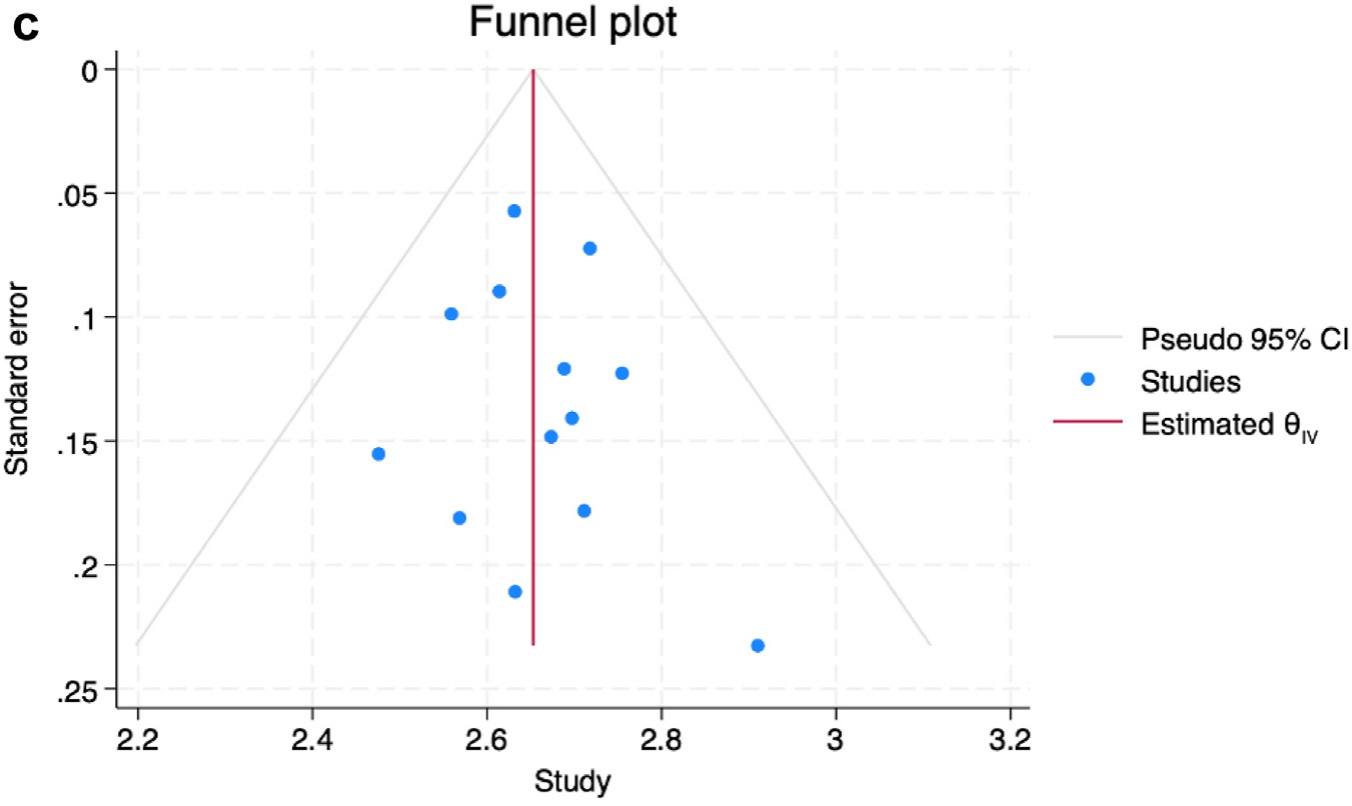
Pooled incidence of SLDA. a. The pooled incidence of SLDA across 12 studies was 1%. b. Procedural success rates with MR <2+ across 13 studies was 95%. c. Funnel plot assessing potential publication bias. Abbreviations: CLASP, Edwards PASCAL TrAnScatheter Valve RePair System Pivotal Clinical; MR, mitral regurgitation; SLDA, single leaflet device attachment; REML, Restricted Maximum Likelihood.

Our analysis demonstrated a low incidence of SLDA and a high procedural success rate with the PASCAL device. The incidence of SLDA with the PASCAL system was comparable to those reported for newer-generation MitraClip devices.^4^ This can be attributed to several factors, including key design features of the PASCAL device—such as its central spacer, contoured large paddles, and nitinol construction, which help seal the regurgitant orifice while minimizing annular deformation and leaflet stress. Additionally, increased operator experience with TEER, as well as advancements in 3D echocardiography, (live multiplanar reconstruction, versatile cropping tools, and enhanced image rendering) have further improved both preprocedural planning and intraprocedural outcomes of TEER.^5^

This meta-analysis has multiple limitations. Firstly, we included diverse study designs with variable follow-up durations and baseline characteristics, contributing to the high interstudy heterogeneity. Most patients underwent TEER with the PASCAL P10 for secondary MR, which may limit generalizability to patients with primary MR. In addition, individual study sizes were small, and although funnel plot assessment did not indicate significant publication bias, the small number of SLDA events currently reported means that unpublished data or negative findings from smaller centers could potentially influence the true incidence of SLDA. Although the observed SLDA rate with the PASCAL device appears similar to previously reported rates with the MitraClip device,^4^ these findings are based on different study populations, therefore direct comparisons between devices should be interpreted with caution. These limitations underscore the necessity for further research to offer more comprehensive insights into SLDA incidence and procedural success across various patient subsets undergoing TEER for MR.

## Ethics Statement

This manuscript adheres to the relevant ethical guidelines.

## Funding

This work was supported by 10.13039/100000968AHA's Second Century of Science Clinical Fellow Research Education Grant.

## Disclosure Statement

The authors report no conflict of interest.

## References

[1] Otto C.M., Nishimura R.A., Bonow R.O. (2021). 2020 ACC/AHA Guideline for the management of patients with valvular heart disease: a report of the American College of Cardiology/American Heart Association joint committee on clinical practice guidelines. Circulation.

[2] Freeman M.F., Tukey J.W. (1950). Transformations related to the angular and the square root. Ann Math Stat.

[3] Higgins J.P.T. (2003). Measuring inconsistency in meta-analyses. BMJ.

[4] Bhatia K., Gupta S., Carter K. (2024). Single-leaflet device attachment after mitral transcatheter edge-to-edge repair. JACC Cardiovasc Interv.

[5] Schneider L., Markovic S., Mueller K. (2022). Mitral valve transcatheter edge-to-edge repair using MitraClip or PASCAL: a multicenter propensity score–matched comparison. JACC Cardiovasc Interv.

